# Structural and computational analysis of peptide recognition mechanism of class-C type penicillin binding protein, alkaline D-peptidase from *Bacillus cereus* DF4-B

**DOI:** 10.1038/srep13836

**Published:** 2015-09-15

**Authors:** Shogo Nakano, Seiji Okazaki, Erika Ishitsubo, Nobuhiro Kawahara, Hidenobu Komeda, Hiroaki Tokiwa, Yasuhisa Asano

**Affiliations:** 1 Biotechnology Research Center and Department of Biotechnology, Toyama Prefectural University, 5180 Kurokawa, Imizu, Toyama 939-0398, Japan; 2 Asano Active Enzyme Molecule Project, ERATO, JST, 5180 Kurokawa, Imizu, Toyama 939-0398, Japan; 3 Research Center of Smart Molecules, Rikkyo University, Nishi-ikebukuro, Toshimaku, Tokyo 171-8501, Japan

## Abstract

Alkaline D-peptidase from *Bacillus cereus* DF4-B, called ADP, is a D-stereospecific endopeptidase reacting with oligopeptides containing D-phenylalanine (D-Phe) at N-terminal penultimate residue. ADP has attracted increasing attention because it is useful as a catalyst for synthesis of D-Phe oligopeptides or, with the help of substrate mimetics, L-amino acid peptides and proteins. Structure and functional analysis of ADP is expected to elucidate molecular mechanism of ADP. In this study, the crystal structure of ADP (apo) form was determined at 2.1 Å resolution. The fold of ADP is similar to that of the class C penicillin-binding proteins of type-AmpH. Docking simulations and fragment molecular orbital analyses of two peptides, (D-Phe)_4_ and (D-Phe)_2_-(L-Phe)_2_, with the putative substrate binding sites of ADP indicated that the P_1_ residue of the peptide interacts with hydrophobic residues at the S_1_ site of ADP. Furthermore, molecular dynamics simulation of ADP for 50 nsec suggested that the ADP forms large cavity at the active site. Formation of the cavity suggested that the ADP has open state in the solution. For the ADP, having the open state is convenient to bind the peptides having bulky side chain, such as (D-Phe)_4_. Taken together, we predicted peptide recognition mechanism of ADP.

Some penicillin-binding proteins (PBPs) take part in bacterial cell wall synthesis by catalyzing transglycosylation and transpeptidation of peptidoglycan^1^. Inhibition of PBPs prevents formation of the cell wall, which is often fatal for bacteria^2^. β-Lactam compounds, such as penicillin and cephalosporin, inactivate PBPs by binding to the active site; therefore, these compounds are potential antibiotics^2^,^3^. However, Poole suggested that overuse of β-lactam antibiotics may have led to the evolution of pathogens resistant to these drugs; several PBPs may have acquired β-lactamase activity that can degrade β-lactam antibiotics^4^. Structural analysis of PBPs could elucidate how β-lactamase activity may have been acquired during the evolutionary process, and this knowledge would facilitate the design of new antibiotics. Many PBP structures have been determined previously^5^,^6^,^7^,^8^,^9^,^10^,^11^,^12^,^13^, and these enzymes can be divided into three classes (A, B, and C) based on differences in their structures and active sites^1^.

In this study, we used alkaline D-peptidase from *Bacillus cereus* DF4-B (ADP), an AmpH-type class C PBP^14^,^15^,^16^, as a model for determining the peptide ligand recognition mechanism of PBPs. ADP acts as a D-stereospecific endopeptidase^14^ and degrades D-phenylalanine tetrapeptide [(D-Phe)_4_] into dipeptides [(D-Phe)_2_]^14^. Some tripeptides can also be degraded by this enzyme to one dipeptide and an amino acid. Tripeptides with a hydrophobic residue at the C-terminal residue are degraded by ADP. On the other hand, the dipeptides produced by the reaction always have D-Phe at the C-terminus^14^. These results suggested that reactive peptides have D-Phe at the acyl donor site (P_1_; detailed explanations about the nomenclature of Schechter and Berger will be described later) and a hydrophobic residue at the acyl acceptor site (P_1_’). In addition, peptides with more than two residues at the acyl donor or acceptor site should be reactive with ADP. Although the exact physiological function of ADP is not determined, the wide distribution may indicate the important role of the D-stereospecific endopeptidase^17^.

ADP also catalyzes synthesis of peptides: for example, it can synthesize D-Phe oligopeptides from D-phenylalanine methyl ester^18^. Wehofsky *et al.* succeeded in using ADP to synthesize proteins entirely composed of L-amino acids, using L-amino acid 4-guanidinophenyl esters (OGp) as acyl donors and amino acid amides and peptides of the L-configuration as acyl acceptors^19^. The OGp leaving group is recognized as D-Phe (P_1_) at S_1_ site of ADP, and enables ADP to mediate the acceptance of originally poorly reactive acyl moieties composed of L-amino acids. These special peptide esters are termed “substrate mimetics”. To date, however, the peptide recognition mechanism of ADP had remained unknown because the crystal structure of the protein was unsolved. Elucidation of the peptide ligand recognition mechanism could facilitate improvements in ADP function, making the enzyme suitable for synthesis of various peptides and proteins.

In this study, we determined the crystal structure of ADP (apo). In addition, we performed molecular dynamics (MD) simulations and first-principles calculations using the fragment molecular orbital (FMO) method^20^. Based on the results, we propose a molecular mechanism by which ADP recognizes peptide ligands containing D-Phe.

## Results

### Crystal structure analysis of ADP

**Figure 1:**
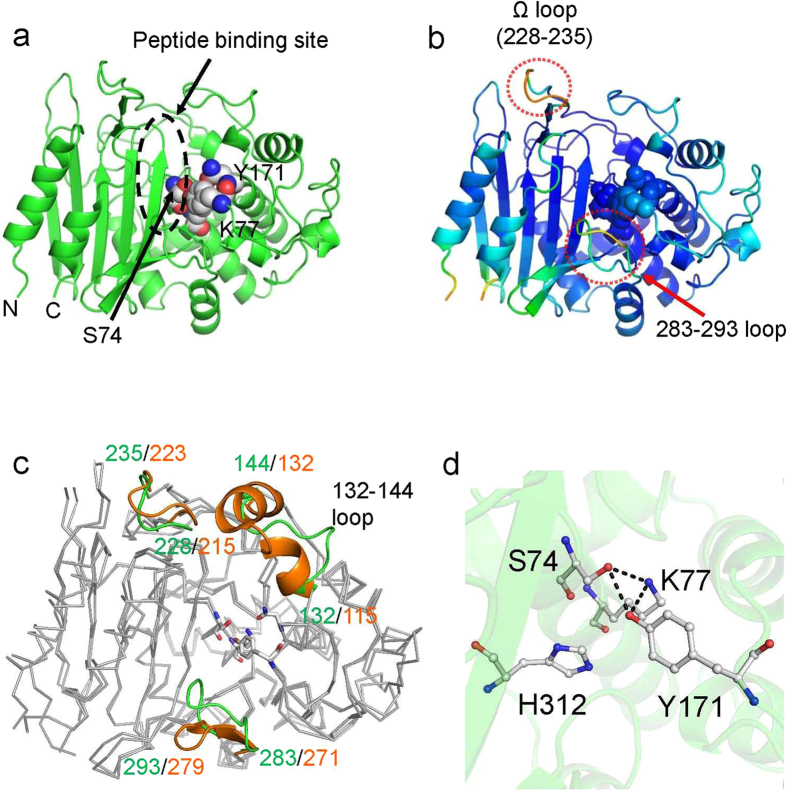
Crystal structures of ADP (apo) form. (**a**) overall structure of ADP (apo) form. The active site residues are colored by gray sphere. (**b**) Overall structure of ADP (apo) form colored by *B*-factor value. The residues having low and high *B*-factor values are colored by blue and red, respectively. (**c**) Superposed structures of ADP (green) and DD-peptidase (PDB ID: 1IKG, orange). The three loop regions (132–144 loop, Ω loop and 283–293 loop) are shown by cartoon model. (**d**) Catalytic tetrad residues in ADP (apo) form. Potential hydrogen bonds were represented by black dash lines. The bond length is less than 3.4 Å. All of figures in this study are prepared by PyMOL^33^. Detailed methods of purification, crystallization and structure determination of the ADP are written in the Supporting Information. The crystallographic parameters are summarized in Table 1. The coordinates and structure factors have been deposited in the Protein Data Bank (PDB ID: 4Y7P).

The crystal structure of the ADP (apo) form was determined at 2.1 Å resolution (Fig. 1a). The space group is *P*3_2_21, and one ADP molecule is present in each asymmetric unit. The crystal structure of ADP consists of two subdomains: a five-stranded β-sheet flanked by three α-helices, and a cluster of mixed 3_10_-helices and α-helices. A cavity for binding substrate peptides is also present near the active-site residues (Fig. 1a).

To highlight the flexible region in ADP, the structure is colored according to the *B*-factor value of Cα atoms in Fig. 1b. Two regions, the Ω loop and the 283–293 loop (Fig. 1b, red circle), had higher *B*-factor values than the average value for the structure overall (32.2 Å^2^): the values for Ω loop and 283–293 loop are 73.9 and 56.7 Å^2^, respectively.

Proteins with structures similar to that of ADP were picked up using the DALI server^21^. The results indicated that ADP is similar to AmpH-type class C PBPs^1^, such as DD-peptidase (PDB ID 1IKG; *Z*-score 48.7, r.m.s.d. of 1.3 Å based on 329 of 345 C^α^ atoms, 36% identity^7^) and D-amino acid amidase (DAA) (PDB ID 2DNS; *Z*-score 42.9, r.m.s.d. of 2.1 Å based on 325 of 361 C^α^ atoms, 29% identity^9^). Therefore, in this study, the ADP structure was compared with DD-peptidase (Fig. 1c). Three regions, the 132–144 loop, Ω loop, and 283–293 loop, have different conformations in the two proteins (Fig. 1c). In particular, the structure of the 132–144 loop of ADP is clearly different from that of DD-peptidase: in ADP, this region consists of 13 amino acids and has a loop structure (Fig. 1c, green), whereas in DD-peptidase it consists of 18 amino acids and contains a helix-turn-helix motif (Fig. 1c, orange). This difference may explain why ADP has both D-endopeptidase and β-lactamase activities; Previously, another group suggested that “loop remodeling,” as observed here for the 132–144 loop, contributes to divergence of enzyme function^22^.

Most AmpH-type class C PBPs contain the three active-site sequence motifs: SXXK, YXN, and HXG^1^. Residues from these motifs form a Ser/Tyr/His/Lys catalytic tetrad^13^ that dominates the catalysis of AmpH-type class C PBPs. This tetrad is also present in ADP (Ser74/Tyr171/His312/Lys77; Fig. 1d). The catalytic tetrad of ADP is conserved in AmpH-type class C PBPs such as DD-peptidase^7^ and DAA^9^, and an oxyanion hole formed by the backbone amide N atoms of Ser74 and Ser315 is also present.

Some differences in substrate specificity between ADP and DD-peptidase^7^ can be explained on the basis of their structural differences. Trp233 of DD-peptidase, which creates a small hydrophobic space required for accommodating a part of the side chain of D-Ala (P_1_) of the substrate (glycyl-L-α-amino-ε-pimelyl-D-Ala-D-Ala [REX]), is replaced by Met245 in ADP; this replacement creates the hydrophobic space required to accommodate a part of the D-Phe (P_1_) side chain of the ADP substrate. Arg285 of DD-peptidase, which recognizes the C-terminal D-Ala (P_1_’) carboxylate group of REX, is replaced by Tyr299 in ADP; this replacement create partial space for the S_2_’ subsite containing the C-terminal carboxylate group of the D-Phe (P_2_’) of the ADP substrate (D-Phe)_4_. Thr299 and Thr301 in DD-peptidase, which create a small hydrophobic space required to accommodate a part of the side chain of D-Ala (P_1_’) of REX, are replaced by Gly313 and Ser315 in ADP, respectively; these replacements create the hydrophobic space required to accommodate a part of the side chain of D-Phe (P_1_’) of (D-Phe)_4_.

### Docking simulation of ADP with two tetra peptides: (D-Phe)_4_ and (D-Phe)_2_-(L-Phe)_2_

**Figure 2:**
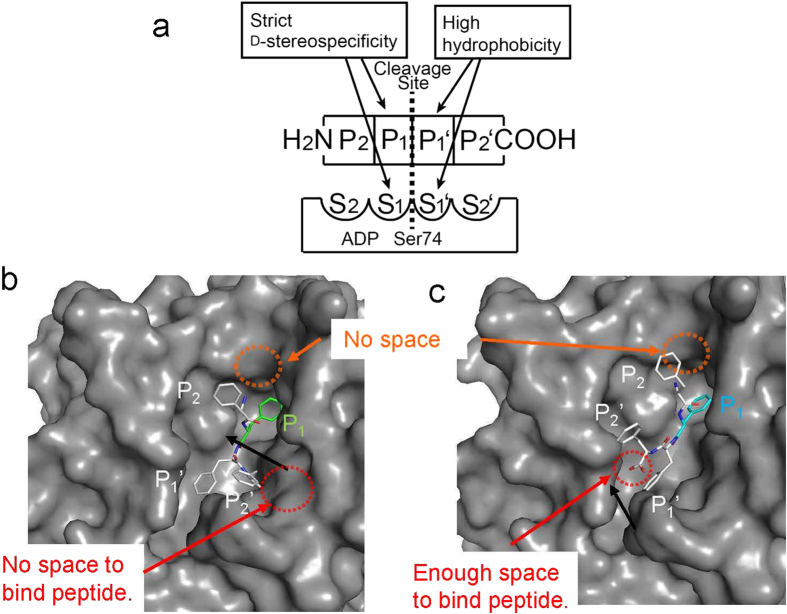
Docking analysis of ADP and peptide ligands. (**a**) Schematic representation of enzyme-substrate complex of ADP with a tetrapeptide. The nomenclature (S, P) is based on that of Schechter and Berger^23^. The active site of the enzyme is composed of subsites (S_2_, S_1_, S_1_’, S_2_’) located on both sides of the catalytic residue Ser74. (**b**,**c**) Docking structure of ADP with (D-Phe)_4_ (**b**) and (D-Phe)_2_-(L-Phe)_2_ (**c**). The docked peptide ligands are shown by sphere model.

Based on the nomenclature of Schechter and Berger^23^ and the fact that ADP catalyzes cleavage of a tetrapeptide into two dipeptides^14^, peptide ligands and their binding sites on ADP can be categorized as shown in Fig. 2a. P_1_ and P_1_’ denote peptide ligand residues on the acyl and leaving groups, respectively, of the scissile bond (Fig. 2a). Adjacent peptide residues are numbered in increasing order (Fig. 2a). The residues of the peptide ligands bind to specific sites within ADP, represented as “S”: P_1_ and P_1_’ residues bind to S_1_ and S_1_’, respectively (Fig. 2a). Biochemical assays of ADP suggested that this enzyme recognizes D-Phe exclusively at the S_1_ site^14^, and favors hydrophobic residues at the S_1_’ site^19^.

To confirm the binding mechanism described above, we tried to solve the structure of peptide-bound ADP. However, despite many attempts using soaking and co-crystallization methods, we could not obtain crystal structures of wild-type or inactivated ADP bound to substrate (D-Phe)_4_. To compensate for the lack of such structures, we performed docking simulations of the peptide ligands (D-Phe)_4_ and (D-Phe)_2_-(L-Phe)_2_ with ADP in the protonated state at pH 7.0, near the crystallization condition. At neutral pH, the side chains of Lys77 and Tyr171 were protonated, the side chain of His312 was in neutral imidazole form, and the N and C termini of these ligands were protonated and deprotonated, respectively. The protonated states of the active-site functional groups are important for enzyme activity because either Lys77 or Tyr171 must be in the basic form to provide a general base for catalysis. From this perspective, the pH conditions used in this study (pH = 7.0) were disadvantageous for enzyme activity. Consistent with this, the relative activity at pH 7.1 was only 7.9% when the observed activity at pH = 10.3 was defined as 100%^14^. The docking structures of ADP with the two peptide ligands are shown in Fig. 2b [(D-Phe)_4_] and 2c [(D-Phe)_2_-(L-Phe)_2_]. Differences between the two structures can be observed in the C-terminal carbonyl groups of the peptide ligands. In (D-Phe)_4_–bound form, the group is oriented toward the interior of the ADP molecule; there is no space to bind another peptide at the C-terminus of the peptide ligand (Fig. 2b, red circle). On the other hand, for the (D-Phe)_2_-(L-Phe)_2_–bound form, the C-terminal carbonyl group is oriented toward the exterior of the ADP, and there is enough space to bind other peptides (Fig. 2c, red circle). At the N-terminal amide group of the peptide ligands, there is no space to bind other peptides, and the residues in the 132–144 and Ω loops interact with each other (Fig. 2b and c, orange circle): in spite of peptide ligands composed of more than four residues, such as (D-Phe)_6_, could bind to ADP^14^,^19^. This interaction may be a result of artificial crystal packing. In fact, Lys144 on the 132–144 loop makes contact with a neighboring molecule that is related by an intermolecular packing interaction via the symmetry operation (-X-1, -Y, -Z-2/3). Similar crystal packing is also observed in the structure of DD-peptidase in complex with REX^7^. It remains possible, however, that the docked models of the ligands in this study differ from the real structures because of the effects of artificial crystal packing. In order to propose a more accurate ligand binding model, it will be necessary to successfully determine the crystal structures of ADP in complex with ligands.

### Interaction energy analysis between ADP and peptides

ADP appeared to recognize peptide ligands by forming hydrophobic and π-π interactions, implying that energy analysis based on first-principles calculation is required to assign the residues of ADP that form stable interactions with peptides. In this study, we performed interaction energy analyses, using the FMO method at the level of the correlated RI-MP2/cc-pVDZ^24^, to obtain the docking structures.

**Figure 3:**
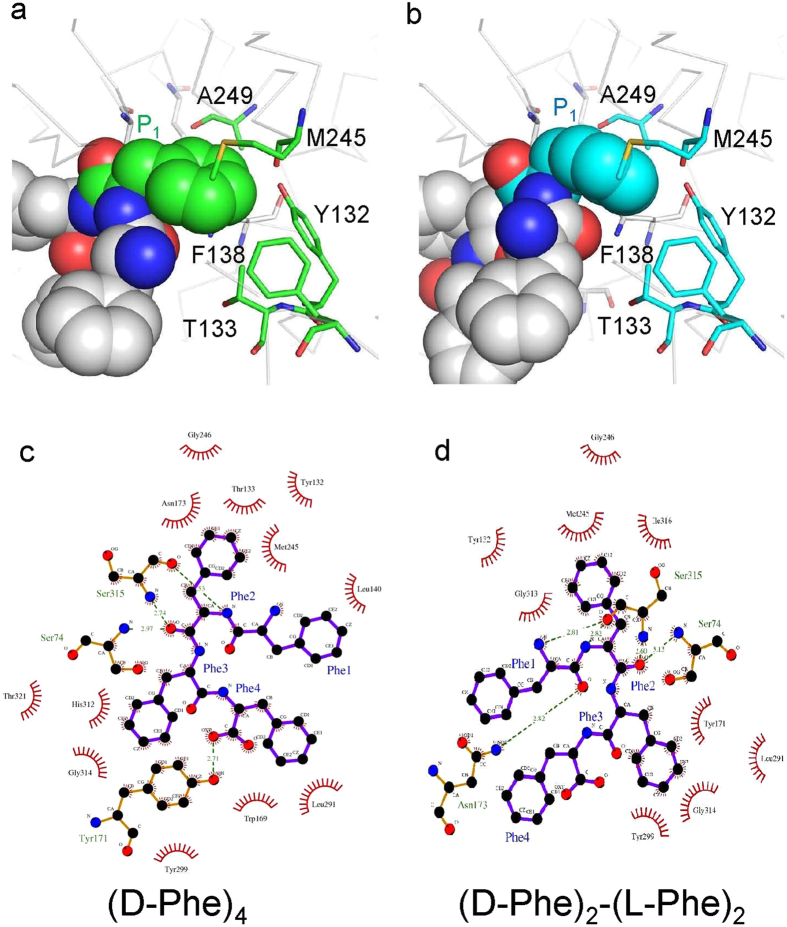
Interaction among P_1_-and S_1_-site residues in the docking structures of (D-Phe)_4_ (**a**) and (D-Phe)_2_-(L-Phe)_2_ (**b**). The potential interacting residues of S_1_ site are shown as green (**a**) and cyan (**b**) stick models. 2D schematic interaction model between ADP and peptide ligands, (D-Phe)_4_ (**c**) and (D-Phe)_2_-(L-Phe)_2_ (**d**). N, C and O atoms are represented by blue, black, and red spheres, respectively. The hydrogen-bond interactions are shown as a dashed lines (green). Interacting atoms are shown as radiating spokes. These figures were prepared utilizing LigPlot+^41^.

The resultant docking structures are energetically favorable; therefore, the docking simulation could be performed correctly: the theoretical binding energies of ADP with (D-Phe)_4_ and (D-Phe)_2_-(L-Phe)_2_ were −120.8 and −71.0 kcal/mol, respectively. These energy values indicated that (D-Phe)_4_ bound more stably to the binding site in ADP than (D-Phe)_2_-(L-Phe)_2_. To elucidate the difference between (D-Phe)_4_ and (D-Phe)_2_-(L-Phe)_2_, the interaction energies were also calculated between the residues in ADP and the peptide ligands. Correlated FMO calculations can correctly evaluate not only electrostatic (hydrophilic) but also van der Waals (hydrophobic) interaction energies between each amino-acid residue in an enzyme and its ligand^25^. Five residues in the S_1_ site are shown in Fig. 3a [(D-Phe)_4_] and 3b [(D-Phe)_2_-(L-Phe)_2_], illustrating the strict recognition of the side chain of D-Phe (P_1_) of peptide ligand. The electrostatic and van der Waals interaction energies of the D-Phe (P_1_) of (D-Phe)_4_ with Tyr132 and Phe138 were −2.81 and −4.14 kcal/mol, respectively, whereas those of (D-Phe)_2_-(L-Phe)_2_ with Tyr132 and Phe138 were −1.79 and −1.31 kcal/mol, respectively. These computational results clearly show that the aromatic residues Tyr132 and Phe138 can form stable interactions with the P_1_ site *via* π-π stacking due to both electrostatic and van der Waals interactions (Fig. 3a,b). The van der Waals attraction energies of the P_1_ site D-Phe of (D-Phe)_4_ with these two residues were significantly larger than those of (D-Phe)_2_-(L-Phe)_2_. The van der Waals interaction energies of the P_1_ site D-Phe of (D-Phe)_4_ with Thr133, Met245, and Ala249 were −2.20, −4.94, and −1.40 kcal/mol, respectively, whereas those of (D-Phe)_2_-(L-Phe)_2_ were −2.87, −1.15, and −0.31 kcal/mol, respectively. Three of the five residues belong to the 132–144 loop, and they might be able to spherically intact with P_1_ site by the ligand-induced fitting. Our computational simulations implied that the induced-fitting effect of the 132–144 loop by D-Phe (P_1_) of (D-Phe)_4_ might be larger than that of (D-Phe)_2_-(L-Phe)_2_. More detailed studies will be required to determine whether this effect influences substrate specificity^14^.

We also estimated the interaction of the peptide ligands from the 2D schematic model of (D-Phe)_4_- (Fig. 3c) and (D-Phe)_2_-(L-Phe)_2_- (Fig. 3d) bound forms. Here, Phe1, Phe2, Phe3, and Phe4 correspond to P_2_, P_1_, P_1_’ and P_2_’, respectively (Fig. 3c,d). In both structures, Phe2 and Phe3 appeared to form many interactions with ADP (Fig. 3c,d): the interactions formed in S_1_ and S_1_’ sites would be important for binding the peptide ligands to ADP. On the other hand, almost no interactions formed at the P_2_ site of (D-Phe)_4_ (Fig. 3c) or the P_2_ and P_2_’ sites of (D-Phe)_2_-(L-Phe)_2_ (Fig. 3d): peptide recognition was less strict at the S_2_ and S_2_’ sites (here, S_2_’ refers to the case in which P_1_’ is L-Phe) than at the S_1_ and S_1_’ sites. Thus, ADP can bind peptides with no (D-Phe) residue at P_2_ and P_2_’ (here, P_2_’ refers to the case in which P_1_’ is L-Phe); several such peptides could react with ADP^14^.

### Molecular dynamics simulation of ADP

As mentioned in the previous section, ADP may adopt different structures in crystal form and solution. To predict the structure in solution, we performed a molecular dynamics (MD) simulation for 50 nsec. Terada *et al.* showed that MD simulation can be utilized to eliminate crystal packing effects and predict protein structure in solution^26^.

**Figure 4:**
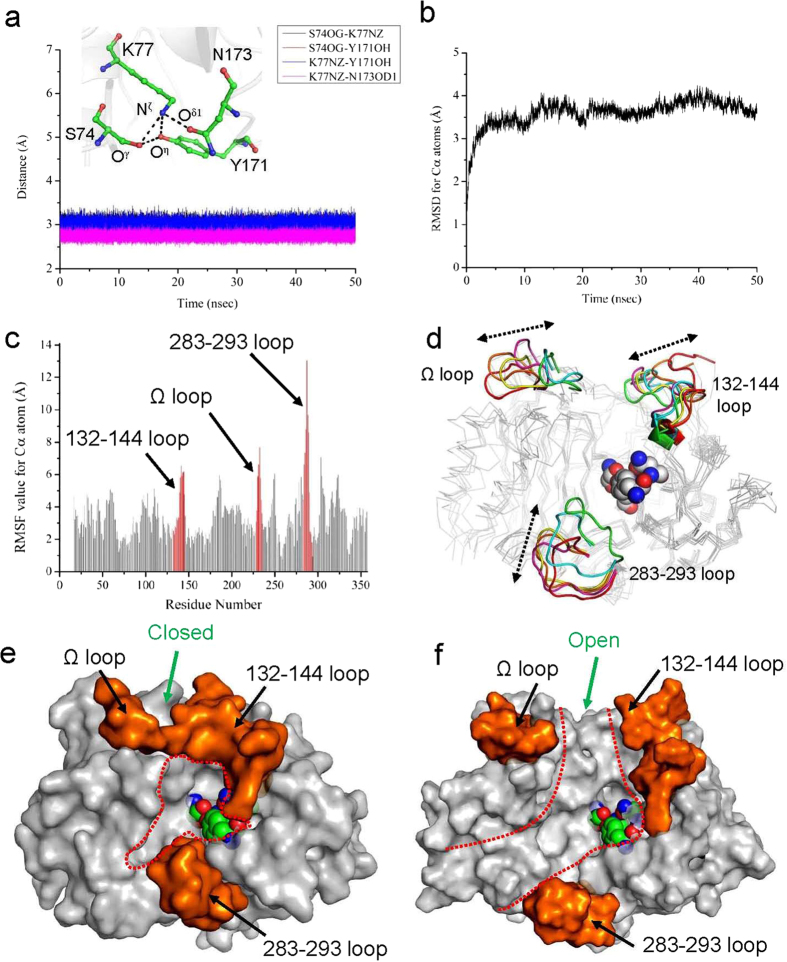
Molecular dynamics simulation of ADP (50 nsec). (**a**) Time-dependent distance change of catalytic tetrad residues. Residue and atom names are indicated in inserted figure. (**b**) Plots of time-dependent change of RMSD value for C^α^ atoms of ADP. (**c**) Plots of RMSF values for C^α^ atoms of each residues. The RMSF value was calculated utilizing total 50,000 structures in the trajectory. The three loop regions (132–144 loop, Ω loop and 283–293 loop) are highlighted in red color. (**d**) Structural change of the three loop regions during the MD simulation. The loop regions are shown in cartoon representation. Trajectory structures at 0, 10, 20, 30, 40, and 50 nsec are colored in green, cyan, magenda, yellow, red and orange, respectively. (**e**,**f**) Surface representation of initial (**e**) and final (**f**) state of the ADP. The residues of the three loop regions are colored by orange. The cavity at the active site was shown in red dotted line. All of trajectory analysis was performed utilizing software named Wordom^40^.

AmpH-type class C PBPs form a catalytic tetrad through hydrogen-bond interactions^7^,^8^,^9^. To accommodate this, we constrained the catalytic tetrad residues of ADP (S74, K77, Y171 and N173) during the simulation. These residues continued to form hydrogen-bond interactions: the average distance values of O^γ^(S74) − N^ζ^(K77), O^γ^(S74) − O^η^(Y171), N^ζ^(K77) − O^η^(Y171), and N^ζ^(K77) − O^δ1^(N173) were 2.99, 2.91, 2.97, and 2.74 Å, respectively (Fig. 4a). The root-mean-square deviation (RMSD) value equilibrated at ~4 Å (Fig. 4b). Taken together, these data indicate that the simulation could be performed correctly.

The flexible region of the ADP structure was assigned based on analysis of the root-mean-square fluctuation (RMSF) value (Fig. 4c) and trajectory structures (Fig. 4d). The calculated RMSF value suggested that the three loop regions, which adopt different conformations in ADP and DD-peptidase (Fig. 1c), had more flexible structures than other regions (Fig. 4c): the average RMSF values for the 132–144, Ω, and 283–293 loops were 4.28, 5.11, and 6.37 Å, respectively. These values were higher than that of the overall structure (3.31 Å). Comparison of five structures in the trajectory (0, 10, 20, 30, 40, and 50 nsec) indicated that the three loop regions also moved flexibly in the direction shown by the dashed arrow in Fig. 4d.

The surface structures of the initial (Fig. 4e) and final (Fig. 4f) states illustrate the structural change that ADP undergoes. Initially, ADP forms a closed state in which the 132–144 and Ω loops interact (Fig. 4d). By contrast, the final state of ADP is open, and the interaction between the two loops is broken (Fig. 4f). The open state favors recognition of bulky peptide ligands by ADP. The importance of this structural change for catalysis was previously described by Shi *et al.*^27^, who suggested that PBP enzymes have the ability to switch between the open and closed states.

## Discussion

**Figure 5:**
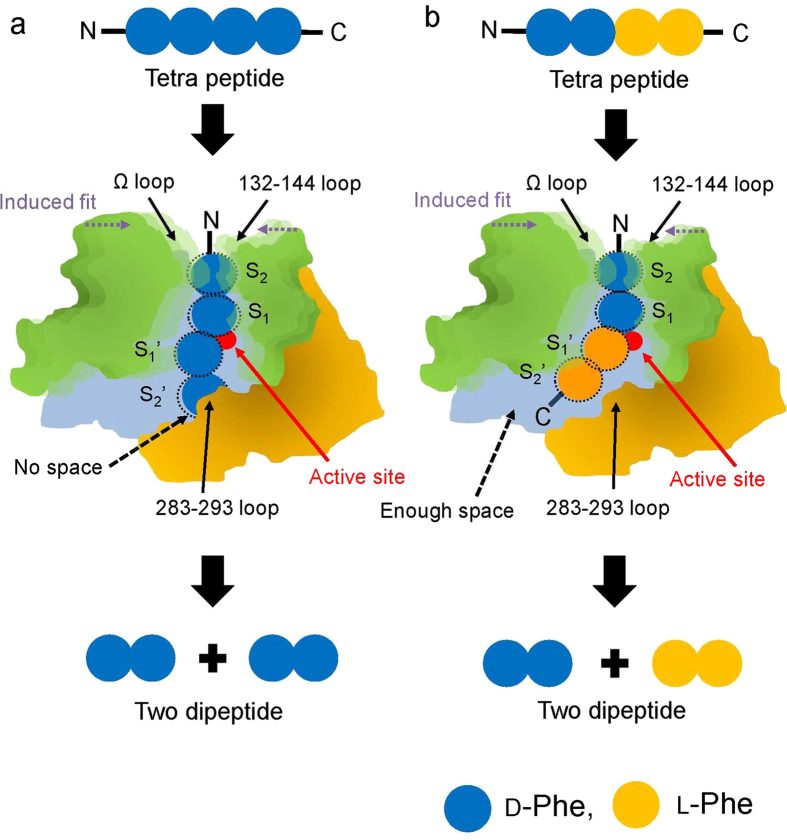
Schematic model of peptide recognition mechanism, (D-Phe)_4_ (a) and (D-Phe)_2_-(L-Phe)_2_ (b), of ADP. In the model, D-Phe and L-Phe are shown as red, blue and orange sphere, respectively. The “N” and “C” in the model represents N-terminal and C-terminal region of the peptides.

Based on the crystal structure, our docking and MD simulation analyses of ADP, and biochemical results obtained in previous studies^14^, we propose a schematic model for recognition of the peptide ligands [(D-Phe)_4_ and (D-Phe)_2_-(L-Phe)_2_] by ADP (Fig. 5). Peptide ligands bound in a cavity at the active site of ADP; the cavity is formed by a flexible structural change involving the 132–144, Ω, and 283–293 loops (Fig. 5). Formation of the large cavity within ADP confers broad specificity toward various peptide ligands^14^,^19^. A previous study reported that size of the cavity affects substrate specificity in PBPs: McDonough *et al.* suggested that the size of the hydrophobic surface at the cavity is important for the broad substrate specificity of β-lactamases^7^. In addition, high flexibility at the loops will affect the reactivity of ADP. The loops may change their structure by induced fit when peptide ligands bind to ADP. In other PBPs, the importance of the flexibility at the loops has been described previously: in DAA, the Ω loop plays an important role in deacylation of substrate^9^, and the flexibility of the Ω loop is one of the factors enabling efficient degradation of third-generation cephalosporins by β-lactamases^2^. The formation of a large cavity and the flexibility of the loops may confer both D-stereospecific endopeptidase and β-lactamase activity on ADP^14^.

The open state of ADP provides space at the N-terminal region of peptide ligands (Fig. 5). This space is advantageous for binding of peptides with long N-terminal L- or D- amino-acid residues at the acyl group (denoted by P_x_ in Fig. 2a, x represents number). Biochemical analysis of ADP also supports the idea that this space is formed: the enzyme can react with (D-Phe)_6_^14^ and recognize a peptide composed of ~10 L-amino acid residues and OGp^19^; the OGp is recognized as D-Phe (P_1_) at the S_1_ site of ADP.

On the other hand, the structure of the peptide C-terminal region clearly differs between the (D-Phe)_4_– and (D-Phe)_2_-(L-Phe)_2_–bound forms. In the former form, C-terminal carboxyl group of the peptide ligand directs toward protein interior, and there is a space to bind only two D-Phe (Fig. 5a). On the other hand for the latter form, there is enough space to bind long L-amino-acid residues (more than two residues) in the peptide ligand (Fig. 5b). This observation suggested that long peptides formed by more than two L-amino-acid residues at the leaving group (denoted by P_x_’ in Fig. 2a, x represents number) could bind to ADP.

To summarize these results, we predict that ADP cleaves the following peptide ligands in a different manner: (D-Phe)_6_ would be degraded to three (D-Phe)_2_, and (D-Phe)_2_-(L-Phe)_4_ would be cleaved to (D-Phe)_2_ and (L-Phe)_4_. This prediction corresponds to the results of a previous study: ADP can bind peptide ligand formed by more than four L-amino-acid residues as the acyl acceptor. In addition, peptide consisting entirely of L-amino-acid residues cannot be cleaved by ADP^19^.

The results of this study should contribute to improvements in the enzymatic function of ADP. However, several points remain to be clarified regarding the reaction mechanism of ADP. For example, in the case of cleavage of peptide ligand by ADP, the energy of the peptide bond must be weakened in order to surpass the energy surface potential, necessitating intermediate reaction states. To elucidate this issue, the energy surface of ADP must be determined via quantum mechanical calculation. Furthermore, the physiological function of ADP remains unknown. We will try to address these questions in the future, using our structural analysis of ADP in this study as a starting point.

## Methods

### Expression and purification of ADP

An *Escherichia coli* transformant expressing ADP was prepared as follows. The *adp* gene, which truncated 25 residues of N-terminal of ADP, was prepared by PCR and was cloned to pET-21b (+) plasmid. Here, SignalP 4.0 software^28^ indicated that 32 of N-terminal residues of ADP was signal peptide, and therefore, we predicted that the truncation does not affect enzyme activity of ADP. In addition, we confirmed activity of the truncated ADP. The utilized restriction enzyme was *Eco*RI and *Xho*I. The *E. coli* host strain BL21 (DE3) was transformed with the resulting plasmid and used for the expression of ADP. The strain was subcultured at 37 °C for 12 h in a test tube containing 5 mL of LB medium supplemented with 100 μg/mL ampicillin. The subculture was then inoculated into 4 L of LB medium supplemented with 100 μg/mL ampicillin and cultured at 37 °C for 10 h. After incubation at 23 °C for 1 h, isopropyl β-D-thiogalactopyranoside was added to the culture at a final concentration of 0.5 mM, and the culture was shaken at 23 °C for overnight. The cells were harvested by centrifugation at 6700 × g for 6 min at 4 °C and washed with buffer A (20 mM potassium phosphate [pH 7.0], 100 mM NaCl, and 10 mM imidazole). The cells were resuspended in buffer A and disrupted with an ultrasonic oscillator. The insoluble fraction was removed by centrifugation (16700 × g, 35 min), and the supernatant was collected and loaded onto a Ni^2+^-Sepharose column. The column was washed with 50 mL of buffer A, and then with 50 mL of buffer A containing 70 mM imidazole, after which ADP was eluted and collected with buffer A containing 300 mM imidazole. The eluted ADP was concentrated and applied to a gel-filtration column (Superdex 200 pg [GE Healthcare, Stockholm, Sweden]) that had been equilibrated with buffer B (10 mM potassium phosphate [pH 7.0], 50 mM NaCl). The presence of ADP in eluted fractions was confirmed by SDS-PAGE, and fractions containing ADP were collected and concentrated for crystallization.

### Crystallization, data collection, structure solution and refinement of ADP

The ADP sample was concentrated to more than 20 mg/mL by Amicon Ultra-15 Centrifugal filter devices (Millipore, Billerica, MA, USA). Protein concentration was calculated by measuring UV-Vis absorbance at 280 nm on a Nanodrop UV-Vis spectrometer (Thermo Fisher Scientific, Waltham, MA, USA). The concentrated sample was mixed with buffer C (30% [w/v] D-glucose) at a sample:buffer ratio of 9:1 for screening of crystallization conditions for ADP. Crystallization was performed by hanging drop vapor diffusion method. Screening of crystallization conditions for ADP was performed using PEG/ION Screening kits (Hampton Research, Aliso Viejo, CA, USA). A 2-μL sample was mixed with 1.0 μL of each reservoir solution from the screening kit. After a 1-week incubation at 4 °C, triangle pole shaped ADP crystals appeared in PEG/ION screen condition 13 (20% [w/v] PEG3350 and 0.2 M sodium thiocyanate).

The crystal was soaked in a cryoprotectant solution (20% [w/v] PEG3350, 0.2 M sodium thiocyanate, 20% (w/v) D-glucose, 1 mM (D-Phe)_4_) for 10 min under 4 °C condition, and then vitrified in liquid nitrogen. The data set was collected in house at 100 K under a stream of liquid nitrogen using a Rigaku Micro-Max007 CuKα rotating anode X-ray generator and a Rigaku R-AXISVII image-plate detector (Table 1). The crystal diffracted to 2.1 Å and 145 frames were collected with a crystal to detector distance being set at 140 mm with 1.0° oscillation steps and 150-s exposure time per frame. Data processing and reduction were carried out using the HKL2000 program suite^29^. Initial phases were determined by molecular replacement using MOLREP^30^, with the crystal structure of DD-peptidase (PDB ID:1HVB) as a template. Model building and structure refinement were performed using COOT^31^ and REFMAC^32^, respectively. All figures were prepared using PyMOL^33^. Crystallographic and refinement parameters are shown in Table 1.

### Peptide docking simulations and FMO analyses

The molecular operating environment (MOE) software^34^ was used for the docking study. The crystal structure of ADP was protonated utilizing Protonate 3D at pH = 7.0, near the crystallization condition, and then charged, followed by the identification of the active site by using the “alpha site finder” module of MOE. Two peptide ligands, (D-Phe)_4_ and (D-Phe)_2_-(L-Phe)_2_, were docked into the crystal structure using the program ASEDock^35^. These procedures consisted of the following four steps: (i) generation of conformations for these ligands using the default conformational sampling method “Conformation Import”; (ii) generation of as ASE model at the active site; (iii) posing and rigid body alignment of these ligands in the ASE model under superposition of the fragment (O=C−N−Cα−C=O) onto the template fragment (O1=C5−N1−C6−C7=O2) of REX in chain A of DD-peptidase (PDB ID 1IKG^7^), which was superposed onto the structure of ADP; and (iv) energy minimization of the posed conformations of these ligands in the concavity. To correctly analyze the interaction energy between the peptide and each amino acid residue of ADP, first-principles (total electronic) calculations, based on the FMO method, were performed for the whole system of the ADP–peptide complex (5332 atoms, 342 amino acid residues of ADP and 4 amino acid residues of peptide). All FMO calculations were carried out using the PAICS software package (http://www.paics.net/index_e.html). In the FMO calculations, polypeptides in ADP-peptide complex were divided into one-residue fragments, with cut-off points at the Cα of each residue. In other words, ADP was divided into 342 fragments, and (D-Phe)_4_ and (D-Phe)_2_-(L-Phe)_2_ were divided into four fragments each.

### Molecular dynamics simulation

Overall structure of ADP (apo) was used as start model for MD simulation. The structure was loaded by software “MOE^34^” and protonated utilizing Protonate3D. Size of cuboid box was set to 95 × 90 × 80 Å, and the TIP3P water molecules were distributed into the box at a solvent density of 1.0 g/cm^3^. The energy minimization was applied to the system by constraining four catalytic residues of ADP; S74, K77, Y171 and N173. Total charge of the system was calculated and sodium (Na^+^) or chloride (Cl^−^) ions were placed to the system to neutralize the charge to zero.

MD simulation was performed using NAMD2.9 software^36^ by applying periodic boundary condition to the system. The Ewald method^37^ was utilized to estimate the electrostatic interactions. The simulation time step was 2 fsec; RATTLE algorithm^38^ was applied to the system by constraining all hydrogens. The pressure and temperature were set to 1.0 bar and 300 K, and Langevin thermostat was adopted to the system.

Prior to start productive MD simulation, simulated annealing procedure was applied to the system performing the same method as shown in previous study^39^. Then productive MD simulation was performed for 50 nsec. In trajectory, the structures were saved every 1 psec. The result was analyzed utilizing trajectory analysis tool, Wordom^40^. During the MD simulation, constrain was imposed to four catalytic residues, S74, K77, Y171 and N173: these residues have to form interaction to express the ADP reactivity.

## Additional Information

**How to cite this article**: Nakano, S. *et al.* Structural and computational analysis of peptide recognition mechanism of class-C type penicillin binding protein, alkaline D-peptidase from *Bacillus cereus* DF4-B. *Sci. Rep.* **5**, 13836; doi: 10.1038/srep13836 (2015).

## Figures and Tables

**Table 1:** Statistics of X-ray diffraction data collection for ADP (apo).

	ADP (apo)
Space Group	*P*3_2_21
Unit cell parameters	
*a* (Å)	104.0
*c* (Å)	98.5
X-ray source	R-AXISVII
Wavelength (Å)	1.54
Resolution (Å)	100–2.10 (2.14–2.10)
No. of reflections^a^	135230
Redundancy	2.3 (2.2)
Completeness (%)	99.9 (100)
I/sig(I)	33.3 (4.5)
*R*_merge_^b^	0.068 (0.487)
*R*^c^	0.174
*R*_free_^d^	0.219
RMSD of geometry	
Bond length (Å)	0.023
Bond angle (degree)	2.119
Geometry	
Ramachandran outlier (%)	0.3
Ramachandran favored (%)	99.7
Average *B* factor (Å)^2^	
Protein atoms	34.4
Thiocyanate atoms	34.6
Water atoms	37.4
PDB code	4Y7P

^a^Sigma cutoff was set to none (F > 0σF).

^b^*R*_merge_ = Σ_*h*_Σ_*i*_|*I*_*i*_(*h*) − <*I*(*h*)>|/Σ_*h*_ *I*(*h*), where *I*_*i*_(*h*) is the *i*th measurement of reflection *h*, and <*I*(*h*)> is the mean value of the symmetry-related reflection intensities. Values in brackets are for the shell of the highest resolution.

^c^*R* = Σ||*F*_*o*_| − |*F*_*c*_||/Σ|*F*_*o*_|, where *F*_*o*_ and *F*_*c*_ are the observed and calculated structure factors used in the refinement, respectively.

^d^*R*_free_ is the *R*-factor calculated using 5% of the reflections chosen at random and omitted from the refinement.
